# Optogenetics in Alzheimer’s Disease: Focus on Astrocytes

**DOI:** 10.3390/antiox12101856

**Published:** 2023-10-13

**Authors:** Elena Mitroshina, Elizaveta Kalinina, Maria Vedunova

**Affiliations:** Institute of Biology and Biomedicine, Lobachevsky State University of Nizhny Novgorod, 23 Gagarin Avenue, 603022 Nizhny Novgorod, Russiamvedunova@yandex.ru (M.V.)

**Keywords:** Alzheimer’s disease, astrocytes, optogenetic, channelrhodopsin, neurodegeneration, Ca^2+^ activity, inflammation

## Abstract

Alzheimer’s disease (AD) is the most common form of dementia, resulting in disability and mortality. The global incidence of AD is consistently surging. Although numerous therapeutic agents with promising potential have been developed, none have successfully treated AD to date. Consequently, the pursuit of novel methodologies to address neurodegenerative processes in AD remains a paramount endeavor. A particularly promising avenue in this search is optogenetics, enabling the manipulation of neuronal activity. In recent years, research attention has pivoted from neurons to glial cells. This review aims to consider the potential of the optogenetic correction of astrocyte metabolism as a promising strategy for correcting AD-related disorders. The initial segment of the review centers on the role of astrocytes in the genesis of neurodegeneration. Astrocytes have been implicated in several pathological processes associated with AD, encompassing the clearance of β-amyloid, neuroinflammation, excitotoxicity, oxidative stress, and lipid metabolism (along with a critical role in apolipoprotein E function). The effect of astrocyte–neuronal interactions will also be scrutinized. Furthermore, the review delves into a number of studies indicating that changes in cellular calcium (Ca^2+^) signaling are one of the causes of neurodegeneration. The review’s latter section presents insights into the application of various optogenetic tools to manipulate astrocytic function as a means to counteract neurodegenerative changes.

## 1. Introduction

Alzheimer’s disease (AD) is a prevailing neurodegenerative condition that is predominantly observed in the elderly population. Its pathogenesis is marked by an aberrant accumulation of β-amyloid (Aβ), resulting from the sequential enzymatic cleavage of the amyloid precursor protein (APP) by β- and γ-secretase enzymes. Notably, the γ-secretase complex’s principal constituents are presenilin (PS) 1 and PS2, mutations of which manifest in the familial variant of AD [1]. Aβ oligomers aggregate within the brain parenchyma, leading to the development of extracellular amyloid plaques. The amyloidogenic hypothesis presently stands as the principal AD causative model and maintains its pivotal status. Notably, it is intriguing that β-amyloid deposition can also manifest in the elderly without concomitant AD. It has been suggested that prior to amyloid plaque formation, prefibrillar oligomers influenced calcium homeostasis and synaptic transmission [2]. 

Aside from β-amyloid accumulation, the most important marker of AD is the presence of neurofibrillary tangles (NFTs), comprising the highly phosphorylated microtubule-associated protein tau (MAPT) [3]. Normally, the tau protein functions to stabilize microtubules and is vital for axonal transport. However, when subjected to hyperphosphorylation by tau kinases, it undergoes structural alterations, rendering it incapable of binding microtubules. The caspase 3 C-terminal truncation of tau and the N-terminal truncation accelerate the aggregation of the unbound tau. The resultant tau oligomers exhibit neurotoxicity, with the ensuing aggregation culminating in the formation of neurofibrillary tangles [4].

The prominent characteristic observed in individuals with AD is the gradual deterioration of cognitive and memory functions, predominantly linked to the depletion of neurons and synapses in the hippocampal formation and neighboring regions [5]. Furthermore, neuroinflammation, impaired mitochondrial function, and oxidative stress increase in the brains of AD patients constitute significant pathogenetic components of this disorder [6].

Among the promising approaches for influencing cells, optogenetics emerges as a notable contender. Optogenetics, a modern technique, amalgamates optical methodologies, genetic engineering, and electrophysiology to regulate specific cellular activities through diverse photosensitive proteins [7]. The application of optogenetics within the brain garners significant interest, given its potential to precisely modulate the activity of specific neuron and astrocyte subsets, thereby offering novel avenues for neurodegenerative disease intervention. Optogenetic studies often employ microbial opsins, light-responsive proteins that can initiate ionic currents, altering the membrane potential in response to different wavelengths of electromagnetic light [8]. Optogenetics encompasses both constructs, enabling cell activation and light-sensitive proteins that can inhibit neuronal activity. Optogenetic tools empower researchers to regulate diverse biological processes within a cell, from intercellular signal transmission, protein expression, and oligomerization to the modulation of gene expression [9]. The hallmark of optogenetics lies in its selectivity, enabling the insertion of light-sensitive transgenic proteins into highly specific cell populations [10]. Consequently, this technique has evolved into a crucial instrument in neurobiology, facilitating the modulation of neural networks governing intricate cognitive and behavioral functions [11,12].

In recent years, optogenetics has seen rapid development, prompting its exploration for in vivo and in vitro applications aimed at modulating nerve cells to address neurodegenerative processes. It has been posited that optogenetics holds the potential for mitigating and potentially reversing neural network dysfunction in AD. Nevertheless, current attempts to manipulate neurons in AD have not yielded groundbreaking success in disease correction. Besides neurons, glial cells, particularly astrocytes, are affected by AD. Astrocytes represent promising candidates for ameliorating nervous system function amidst neurodegeneration. This review centers on contemporary approaches and future prospects regarding the utilization of optogenetic methodologies to restore brain function in AD, with a particular emphasis on the role of astrocytes.

Astrocytes are pivotal homeostatic regulators of the central nervous system (CNS) [13]. They execute numerous essential functions within the brain, including furnishing metabolic substrates to neurons and other glial cells [14], maintaining extracellular ion homeostasis, providing structural support, and participating in neurovascular interactions [13,15,16]. Through ion channels, neurotransmitter receptors, and transporters, as well as intracellular signaling pathways, astrocytes can perceive and integrate neural information [17]. Furthermore, astrocytes play a role in regulating neuronal synaptic plasticity and excitability by releasing neuroactive agents known as gliotransmitters [18]. 

**Figure 1:**
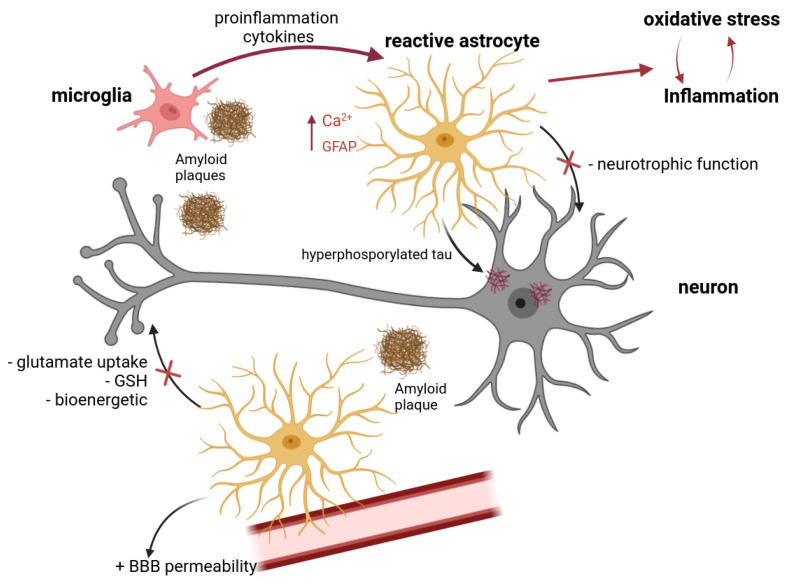
Schematic diagram showing the interaction between different types of cells contributing to the progress of AD pathogenesis. BBB—brain–blood barrier, GSH—glutathione, GFAP—glial fibrillary acidic protein.

Recent investigations have underscored the pivotal involvement of astrocytes in developing neuroinflammation and oxidative stress in AD [19,20], as well as in the synthesis and clearance of amyloid proteins [21]. In AD, various dimensions of astrocytic function suffer impairment, encompassing calcium signaling, the metabolism of glutamate and other neuro- and gliotransmitters, extracellular potassium buffering, and energy metabolism [22]. Schematically, the role of glial cells in AD is shown in Figure 1. 

Hence, astrocytes emerge as highly promising targets for addressing neurodegenerative alterations. The potential therapeutic efficacy of astrocytes in AD, attributable to their involvement in aging, neuroinflammation, the release of neurotrophic factors, and Aβ clearance, has been extensively elucidated [20,23]. The application of optogenetic techniques to activate astrocytes, thereby restoring their functional status and rectifying astrocyte–neuronal interactions, as well as neuron–glial network operations, holds significant promise. In this review, we have endeavored to consolidate existing knowledge regarding the utilization of optogenetics for AD correction, with a specific focus on modulating astrocytic metabolism. An imperative facet is the critical evaluation of translational challenges linked to the potential medical applications of optogenetic tools. Foremost among these are the invasiveness associated with optical fiber implantation for light stimulation and safety concerns concerning the viral delivery of opsin genes. These aspects are comprehensively addressed in the final section of this review.

## 2. Optogenetics as a Tool for Regulating the Activity of Nerve Cells

Optogenetics represents a combination of optical techniques and genetic technologies, harnessing genetically encoded light-sensitive protein-ion channels or opsins, for instance, halorhodopsin, channelrhodopsin, and archaerhodopsin. These opsins induce either cell membrane depolarization or hyperpolarization in response to specific wavelengths of light [24]. By selectively expressing these exogenous photosensitive proteins, it becomes possible to modulate neuronal activity, intracellular signaling pathways, or gene expressions with spatial and temporal specificity. Excitatory opsins can stimulate cellular activity, whereas inhibitory opsins can suppress it. Opsins exhibit variability in their mode of operation and response rates, offering the capability to regulate neuronal activity with remarkable precision.

Optogenetics offers a key advantage in its ability to induce opsin expression within specific cell types. For this purpose, viral vectors are used, that is, the transduction method. Viral vectors are viruses (such as adeno-associated virus (AAV) or lentivirus) that have been modified to carry the desired gene sequence. These modifications render the viral vectors incapable of replication and enhance their safety. By employing promoter sequences specific to neuronal subtypes, opsin expression can be precisely targeted to particular cell types, such as inhibitory or excitatory neurons [25].

The primary families of opsins used in optogenetics include the non-selective cation channel family, which originates from the wild-type Channelrhodopsin 2 (ChR2) initially found in Chlamydomonas reinhardtii. These channels provide a passive flow of protons and positively charged K^+^, Na^+^, and Ca^2+^ ions along an electrochemical gradient when photostimulated with light at a wavelength of approximately 470 nm (blue light) through photoisomerization: the light induces a conformational change in the all-trans-retinal 13-cis configuration. In mammalian neurons, the activation of these channels leads to membrane depolarization and an increased likelihood of generating action potentials. One limitation to the use of these opsins is that blue light possesses a relatively short wavelength and does not deeply penetrate tissues. The utilization of high-intensity light can induce tissue heating, potentially causing unpredictable effects on cell physiology, including tissue damage [26].

Inwardly directed chloride pumps are a family of inhibitory opsins, with some being wild-type variants of Halorhodopsin (HR), originally discovered in Natronomonas pharaonis. These channels are activated by light with a wavelength of 580 nm. When activated, they pump one chloride ion into the cell for each photon of light, elevating the intracellular concentration of Cl^−^ ions. This action results in membrane hyperpolarization, reducing the likelihood of action potential generation [27].

Outward proton pumps also inhibit neuronal activity. Upon activation, these opsins pump protons out of the cell, leading to membrane hyperpolarization. This process also causes an increase in intracellular pH, which is a crucial consideration when designing experiments. Proton pump opsins are derived from natural archaerhodopsin-3 (Arch), originally identified in Halorubrum sodomense [26]. Various modified versions of this type of opsin have been developed, including ArchT [28], eArch3.0, eArchT3.0, eMac3.0 [29], and so on.

Opto-XRs represent a family of opsins associated with GPCRs, including specific variants linked to Gq and Gs proteins. G-protein-coupled receptors indirectly influence neuronal activity by directly activating G-proteins and their downstream targets, thereby regulating signaling cascades [30].

Ongoing research continually develops novel approaches and tools for controlling various aspects of cellular activity. For instance, the photocleavable protein PhoCl is utilized to create light-activated Cre recombinase, the Gal4 transcription factor, and viral protease, which can subsequently activate the Pannexin-1 ion channel [31]. Reference may also be made of the tool Opto-SOS, which enables the light activation of Ras and activates the Ras/Erk kinase pathway [32]; light-inducible transcriptional effectors, LITE, an optogenetic system that integrates the customizable TALE DNA-binding domain with the light-sensitive cryptochrome 2 protein and its interacting partner CIB1 [33]; VP-EL222, a modified version of the bacterial protein EL222 that binds to DNA under blue light [34]; the blue-light-gated potassium channel BLINK1 [35]; and various similar approaches.

**Figure 2:**
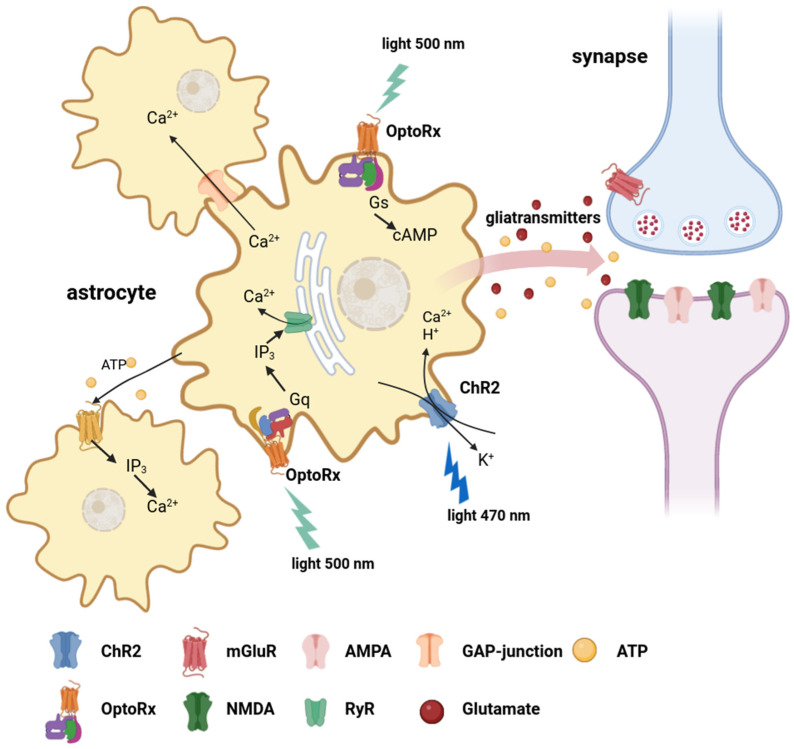
Optogenetic stimulation of astrocytes. A variety of genetically encoded effector molecules for optogenetics have been employed to manipulate intracellular ionic concentrations (H^+^, Na^+^, Ca^2+^, K^+^) and signaling cascades (Gq, Gs, IP3, cAMP) in astrocytes, which evoke the release of gliotransmitters (glutamate, ATP, etc.) and the modulation of synaptic transmission. ChR2—channelrhodopsin-2; OptoXRs—light-driven chimeric G-protein-coupled receptors; NMDAR—N-methyl-D-aspartate receptor; AMPAR—α-amino-3-hydroxy-5-methyl-4-isoxazolepropionic acid receptor; ATP—adenosine triphosphate; IP3—inositol 1,4,5-trisphosphate; cAMP—cyclic adenosine monophosphate; mGluR—metabotropic glutamate receptor; RyR—ryanodine receptor.

Optogenetic technologies have primarily been applied to neurons, but they are evolving and expanding their applicability. The activation of ChR2 in astrocytes can lead to depolarization, intracellular pH reduction, and an increase in [Ca^2+^]. These changes can significantly influence astrocyte activity and modulate the release of gliotransmitters (Figure 2) [36]. 

One notable study demonstrated that by employing ChR2 and the optogenetic stimulation of astrocytes, it was possible to induce an increase in [Ca^2+^] in response to a decrease in pH, which subsequently triggered chemoreceptors in the brainstem through an ATP-dependent mechanism, leading to respiratory responses in vivo [37].

In a highly valuable work, Gerasimov et al. performed a light stimulation of astrocytes expressing either ChR2 (ionotropic opsin) or Opto-α1AR (metabotropic opsin) to determine the effect of activated astrocytes on the functioning of neurons. Together with the stimulation of astrocytes using patch clamps, the activity of pyramidal neurons of the hippocampus was recorded. The activation of ionotropic opsin ChR2 in astrocytes appeared to have a positive effect on the excitability of interneurons but had a negative effect on the activity of pyramidal neurons, leading to a reduction in the frequency of their action potentials. This dual effect might be attributed to variations in light stimulation patterns and neuronal activity states. The stimulation of the metabotropic opsin Opto-α1AR was suggested to enhance long-term synaptic plasticity in mice, which is associated with the secretion of D-serine and glutamate by astrocytes due to an increase in intracellular calcium concentration. When recording spontaneous activity in slices of hippocampal pyramidal neurons, the optimal parameters of optogenetic stimulation were determined [38]. This study holds promise for normalizing synaptic transmission and plasticity in various neuropathologies, including AD.

In a recent study by Maltsev et al. [39], the impact of hippocampal astrocyte activation achieved using two distinct optogenetic tools (the ionotropic AAV-ChR2-mCherry and the metabotropic AAV-Opto-a1AR-EYFP (Gq-linked opsin Opto-a1AR) expressed under the truncated GFAP promoter) on hippocampal neuron activity in mice and rats was investigated using the patch clamp technique. The study elucidated contrasting outcomes associated with these constructs. The activation of ChR2 induced a reduction in the basal field excitatory postsynaptic potentials (fEPSPs) in the CA1 region of the hippocampus following a light stimulation of astrocytic ChR2. Notably, this effect was contingent upon the activity of type 2 purinergic receptors, and the involvement of GABA receptors in mediating these effects was also demonstrated. Conversely, a light stimulation of Opto-a1AR expressed in astrocytes resulted in an augmentation of basal fEPSPs and a significant enhancement in synaptic responses to TBS. This effect was mediated through the activation of the Gq protein and subsequent activation of phospholipase C. Importantly, only the stimulation of Opto-a1AR and not ChR2 elicited the upregulation of early response genes, including cRel, Arc, Fos, JunB, and Egr1 [39]. These findings highlight the capacity of distinct optogenetic constructs expressed in astrocytes to exert opposing effects on synaptic transmission, offering a versatile approach to address a wide spectrum of research inquiries and modulate information processing within the hippocampus.

## 3. The Role of Astrocytes in the Pathogenesis of Alzheimer’s Disease

### 3.1. Production and Clearance of Amyloid Proteins

As per the prevailing hypotheses, β-amyloid (Aβ) assumes a pivotal role in the pathogenesis of AD. While neurons are the primary source of Aβ in the brain, astrocytes are also implicated in its synthesis and processing. Even a minor alteration in Aβ production by astrocytes can exert a notable influence on the amyloid burden. BACE1 secretase cleaves the β site of the amyloid precursor protein (APP) and initiates Aβ production. A study [40] demonstrated that in transgenic mice overexpressing the London mutant of APP [V717I], the focal activation of astrocytes occurs prior to amyloid plaque formation, concomitant with an elevation in BACE1 activity. This astrocytic activation followed an inflammatory A1 pattern [41]. 

Apolipoprotein E (APOE) is a multifunctional protein pivotal in lipid metabolism and neurodegenerative disease development. It is involved in the homeostatic control of plasma and tissue lipids, is produced predominantly by astrocytes and microglia, and has three main isoforms: APOE-ε2, APOE-ε3, and APOE-ε4, among which APOE-ε3 is the most frequently expressed [42]. Nevertheless, inflammation’s presence can impact APOE expression and secretion at both isoform and cellular levels. Notably, human astrocytes bearing the APOE-ε4 allele manifest a pro-inflammatory phenotype featuring heightened nuclear factor (NF-κB) activity [43]. The APOE-ε4 isoform is recognized as a significant genetic risk factor for sporadic late-onset AD, and its role as a biomarker for AD risk is under current investigation [44].

The excessive accumulation of toxic forms of Aβ stems from an imbalance between production and clearance. The removal of soluble Aβ peptides occurs through various mechanisms, such as glial phagocytes, enzymatic degradation, and transport across brain barriers.

Astrocytes transfer amyloid from the brain parenchyma into the cerebrospinal fluid via aquaporin-4-dependent perivascular drainage. Aquaporin 4 (AQP4) is a protein that forms water channels in the cell membrane; it resides predominantly in perivascular endfeet and astrocytic membranes, contributing to CNS water homeostasis. Its densest presence is in astrocytes adjacent to vessels [45,46]. AQP4 knockout mice exhibited a 70% reduction in amyloid elimination [47] and exacerbated cognitive deficits [48].

Beyond the mechanical removal of Aβ from the brain, astrocytes possess the capability to internalize and enzymatically degrade Aβ, as observed in both mouse models in vivo [49], and in primary cultures of mouse astrocytes in vitro [50] as well as human astrocyte cultures [51]. The intracellular degradation of Aβ within astrocytes occurs through lysosomal pathways, while extracellular degradation involves the release of proteases. For instance, astrocytes surrounding amyloid plaques in older mice demonstrated elevated expression of matrix metalloproteinases MMP 2 and 9, which degrade amyloid [52].

### 3.2. Neuroinflammation and Reactive Astrogliosis

In AD, a cascade of pathological processes occurs, including neuroinflammation. Astrocytes are significant contributors to the development of inflammation in neural tissues. They can trigger inflammatory processes by activating various intracellular pathways, such as the NF-κB pathway, the production of nitric oxide (NO) and reactive oxygen species (ROS), as well as the release of several pro-inflammatory cytokines, including IL-1β, IL-6, and TNF [19]. Nevertheless, astrocytes also have the capacity to express and release anti-inflammatory and neuroprotective factors [53], indicating their potential pivotal role in regulating the neuroinflammatory processes observed in AD. Furthermore, the dysregulation of astrocytes contributes to increased cytotoxicity and oxidative stress by promoting amyloid protein production and Aβ plaque aggregation in AD [54]. 

Maladaptive inflammation can be mediated by the complement system (CS), which operates with the involvement of extracellular vesicles (EVs) of neuronal and astrocytic origin. In the brain, EVs facilitate the exchange of molecules between cells and can traverse the blood–brain barrier to enter the periphery [55]. Notably, AD patients exhibited significantly elevated levels of CS components related to the classical and alternative pathways, including C1q, C4b, C3d, factor B, factor D, Bb, C3b, and C5b-C9, as well as the membrane attack complex (MAC). Conversely, the levels of regulatory proteins such as CD59, DAF, and CD56 were reduced in AD patients [56]. When neurons were exposed to astrocyte-derived vesicles obtained from AD patients, it led to an increase in markers associated with necroptosis, suggesting neurotoxicity mediated by the MAC. However, further treatment of these neurons with CD59 prevented their death, which again indicates MAC-mediated toxicity [55,57]. In the astrocytic vesicles from the AD patients, the levels of BACE-1 and sAPPβ were significantly increased, while the levels of GDNF were decreased compared to the control group [58].

An important pathogenetic component of neurodegenerative processes, as well as CNS damage of various etiologies (ischemia, trauma, infection, etc.), is the transition of astrocytes to a state called reactive astrogliosis.

Astrocyte reactivity is initially characterized by soma hypertrophy and the production of pro-inflammatory molecules. Furthermore, there is an increased expression of intermediate filament proteins such as glial fibrillary acidic protein (GFAP), vimentin, and nestin [59]. In AD, there is a notable increase in the population of reactive astrocytes, particularly in the vicinity of amyloid plaques. They are discernible even in the early stages of AD, preceding neuronal degeneration, and persist throughout the progression of the disease [60,61]. Research indicates that the activation of microglia, leading to the release of inflammatory cytokines such as IL-1alpha (IL-1α), IL-1beta (IL-1β), IL-6, tumor necrosis factor-α (TNF-α), and complement component 1q (C1q), induces the formation of reactive astrocytes [62]. Moreover, these cytokines can stimulate the activity of β-secretase and γ-secretase, promoting the cleavage of amyloid precursor protein (APP) and enhancing the production of β-amyloid by astrocytes, thus contributing to the overall load of neuronal β-amyloid [63].

The understanding of astrocyte reactivity has undergone significant evolution in the past decade. Initially, they were regarded as a homogeneous group, but ongoing research is now actively exploring their diverse subpopulations and heterogeneity. In 2021, a comprehensive definition was established, characterizing reactive astrogliosis as a process wherein astrocytes engage in specific molecular programs. These programs encompass alterations in transcriptional regulation, as well as biochemical, morphological, metabolic, and physiological transformations. Ultimately, these changes result in the acquisition of new functions, or the reduction or augmentation of their homeostatic functions, all in response to pathological conditions [64].

**Figure 3:**
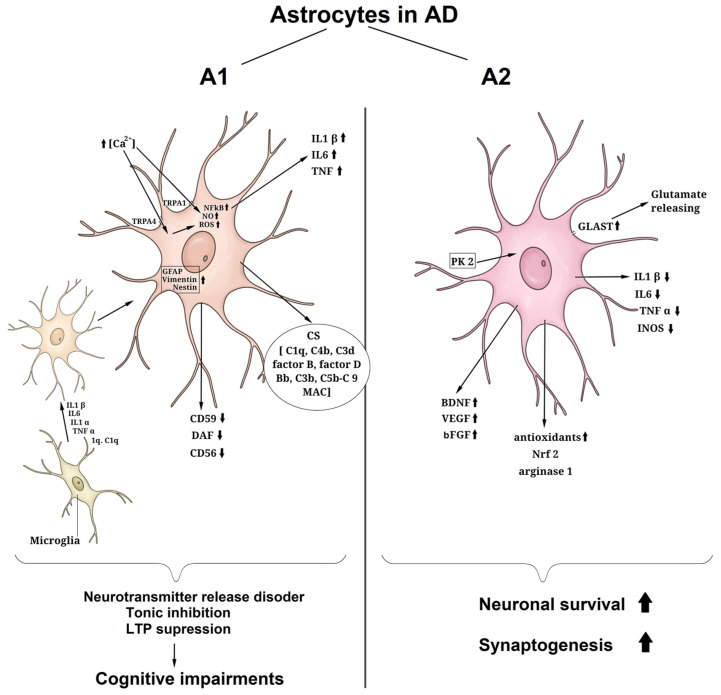
Reactive astrogliosis in neurodegenerative disease and polarization of astrocytes according to the bidirectional (**A1**,**A2**) or neurotoxic–neuroprotective model. BDNF—brain-derived neurotrophic factor, bFGF—basic fibroblast growth factor, CS—complement system, DAF—decay-accelerating factor (CD55), GFAP—glial fibrillary acidic protein, GLAST—glutamate aspartate transporter, IL—interleukin, MAC—membrane attack complex, Nrf2—nuclear factor erythroid 2-related factor 2, PK2—prokineticin 2, ROS—reactive oxygen species, TNF—tumor necrosis factor, TRPA—transient receptor potential ion channels, VEGF—vascular endothelial growth factor. Up arrows—increased expression or concentration of a compound, or increased some process, down arrows—decrease in expression or concentration of a compound or weakening some process.

Reactive astrocytes represent a diverse group of cells whose characteristics are contingent upon the specific conditions and events within CNS pathologies. In an effort to distinguish between various cell phenotypes, reactive astrocytes are commonly categorized into two primary types: A1, which are perceived as potentially destructive and neurotoxic, and A2, which are deemed to support neuron survival and maintenance, thereby possessing neuroprotective qualities (Figure 3) [53,60].

The predominately adverse effects of reactive astrocytes on AD are frequently emphasized. For instance, in the GiD reactive astrocyte model, an excessive generation of hydrogen peroxide by monoamine oxidase B (MaoB) has been observed, subsequently giving rise to tautopathy, glial activation, neuronal death, brain atrophy, and cognitive decline [65]. Furthermore, in an APP/PS1 mouse model, it has been demonstrated that astrocytes located in proximity to amyloid plaques and converted into a reactive form produce elevated quantities of GABA. GABA is released through the bestrophin 1 (Best1) channel and activates neuronal GABAA and GABAB receptors, ultimately suppressing the release of mediators into the synaptic cleft. This resulted in a reduction in the number of spikes in the perforant pathway to the dentate gyrus, impaired synaptic plasticity, and memory deficits [66]. Reactive astrocytes are also implicated in GABA accumulation that culminates in tonic inhibition, the suppression of long-term potentiation (LTP), and consequently, cognitive impairment [67].

The transformation of astrocytes into a reactive state can disrupt the activity of transient receptor potential (TRP) channels, thereby causing disturbances in calcium homeostasis. For instance, in an animal model (APP/PS1 Tg), astrocytes exhibited an overexpression of TRPA1. Through these channels, an Aβ-mediated increase in calcium influx into the cell occurred. Consequently, it stimulated protein phosphatase 2B (PP2B) along with the transcription factors NF-κB and NFAT, ultimately resulting in the production of pro-inflammatory cytokines. The functional loss of TRPA1 had a positive impact on the cognitive abilities of mice. These findings suggest the involvement of astrocytic TRPA1 in Aβ-triggered inflammatory responses in astrocytes and the progression of AD [68]. Additionally, in dissociated cultures of the rat hippocampus, Aβ was found to elevate the expression of TRPV4 and GFAP in astrocytes. This was accompanied by an increase in the intracellular Ca^2+^ levels in astrocytes, the generation of ROS, and significant neuronal damage. However, the inhibition of TRPV4 effectively reduced cell death [69].

Type A2 astrocytes have been comparatively less explored. However, it has been observed that they can reduce extracellular glutamate levels, thus mitigating excitotoxicity. When exposed to prokineticin-2 (PK2), astrocytes adopt the A2 phenotype, leading to increased expression of the glutamate–aspartate transporter (GLAST) and enhanced glutamate uptake by primary mouse astrocytes. This shift is accompanied by a decreased expression of pro-inflammatory factors (IL-1β, IL-6, TNFα, and iNOS) and an increased expression of antioxidant factors (arginase-1 and Nrf2) [70]. A2-reactive astrocytes (A2) are also known to enhance the production of numerous neurotrophic factors (BDNF, VEGF, and bFGF), which promote the survival of neuronal cells and synaptogenesis [60]. Therefore, reactive astrogliosis can exhibit both neuroprotective and potentially disease-promoting effects in AD. The specific signaling pathways involved in the induction of the A1 and A2 phenotypes in CNS damage and neurodegeneration remain to be elucidated.

### 3.3. Oxidative Stress

Oxidative stress arises from an imbalance between the production of free radicals and antioxidants within mitochondria. An excessive production of free radicals, particularly reactive oxygen and nitrogen species, combined with disruptions in the body’s antioxidant enzyme systems, results in the damage of cellular structures, lipids, proteins, and nucleic acids. It is widely acknowledged that oxidative stress is linked to the etiology of nearly all neurodegenerative diseases [71]. Under normal physiological conditions, astrocytes play a critical role in producing antioxidants within neurons. They synthesize and provide amino acids such as glycine and cysteine, which are essential for generating one of the primary antioxidants, glutathione (GSH), in neurons [72]. Additionally, astrocytes are actively involved in synthesizing glutathione and transporting it to neurons through the ABCC1 transporter [73]. The presence of Aβ plaques can influence the antioxidant function of astrocytes. Both in vivo and in vitro studies have demonstrated that monomeric forms of Aβ enhance the expression of ABCC1 during the acute and late stages of AD, which serves as a compensatory mechanism against oxidative stress. However, prolonged exposure to fibrillar forms of Aβ results in reduced ABCC1 transporter expression in the cerebral cortex of 5xFAD mice [72,73]. 

There is substantial evidence indicating a direct correlation between Aβ levels and the production of ROS due to the expression of inducible nitric oxide synthase (iNOS), resulting in nitrosative stress within neurons [74]. Research has demonstrated that the activation of astrocytic iNOS by Aβ is contingent on pro-inflammatory cytokines such as IL-1β and TNF, facilitated through an NF-κB-dependent signaling pathway that triggers NIK kinase. It is possible that the activation of reactive astrocytes according to the neurotoxic and neuroprotective profile correlates with the level of ROS production [20]. 

Reactive oxygen species (ROS) originate from various cellular sources. Notably, enzymes involving cyclooxygenase, lipoxygenase, xanthine oxidase, and cytochrome P450-dependent oxygenases contribute to ROS generation [75]. Additionally, NADPH oxidases (NOX), a distinct group of enzymes solely dedicated to ROS production, warrant mention [76]. In neurovascular units, NOX emerges as a pivotal ROS source [77]. The excessive production of ROS by the NOX family has significant implications, disrupting oxidative homeostasis and contributing to the pathogenesis of neurodegenerative disorders. This enzyme family comprises seven isoenzymes, including NOX1-5, along with two dual oxidases, DUOX1 and DUOX2. NOX is responsible for the formation of superoxide radicals (O^2•−^) by transferring electrons from NADPH through FAD and two heme residues to molecular oxygen. Subsequently, O^2•−^ radicals undergo dismutation to yield H_2_O_2_. It is worth noting that certain isoenzymes, specifically NOX4, DUOX1, and DUOX2, are suggested to directly produce H_2_O_2_. In contrast, Nox5 appears to generate both O^2•−^ and H_2_O_2_ [76,78,79]. Under normal physiological conditions, NOX exhibits low activity. Nevertheless, NOX2-derived ROS are recognized for their regulatory roles in vasodilation, immune responses, and microglial activation [80,81].

In AD, notable morphological and functional cerebrovascular disorders are observed in the brain. These include microvascular atrophy, degradation of the basement membrane, and the deposition of proteoglycans such as heparin sulfate, collagen IV, and laminin. Additionally, there is a reduction in the density of the cerebrovascular network, accompanied by alterations in endothelial cell function. This collective disruption results in hypoperfusion, hypoxia, chronic inflammation, and compromised permeability of the blood–brain barrier (BBB). These vascular abnormalities are collectively referred to as cerebral amyloid angiopathy (CAA), which is closely associated with ischemic lesions, micro- and macrohemorrhages, and impaired cerebral blood flow [82]. Notably, oxidative stress plays a pivotal role in augmenting the permeability of the endothelial monolayer.

Recent research has highlighted the significance of NADPH oxidase activation in the development of AD. This is supported by post-mortem investigations revealing the translocation of NOX2 subunits, specifically p47phox and p67phox, from the cytosol to the membrane. This translocation is presumed to occur within activated microglia [83,84]. Notably, the vessels within the two primary regions most affected in AD, namely the cerebral cortex and the hippocampus, exhibit significantly elevated levels of intracellular O^2•−^, along with increased levels of both Nox 2 and 4 [85]. Additionally, in the frontal lobe of AD patients, especially in the early stages, high mRNA levels of NOX1 and NOX3 have been observed. This suggests the potential involvement of other NOX isoforms in the neuropathology of AD [86]. Microglial NOX2 activation can be triggered by various factors, including Aβ42. Proinflammatory cytokines, chemokines, and NOX2-derived ROS collectively contribute to further microglial activation and neuronal damage, thereby establishing a link between inflammation and oxidative harm in the context of AD pathology [87].

NADPH oxidase 1 has been demonstrated to play a pivotal role in mediating amyloid β-induced damage to the endothelial barrier [88]. Similarly, the involvement of NOX2 in the generation of reactive oxygen species, ultimately leading to the disruption of BBB integrity, has been substantiated. Notably, the application of NOX-2 inhibitors has shown significant reductions in these detrimental effects [89].

Aβ deposition within vessel walls seems to initiate a detrimental cycle: Aβ elevates levels of ROS, which, in turn, enhances Aβ deposition. This concept underpins the hypothesis of AD development, proposing that cerebrovascular injury serves as the initial trigger for neuronal damage and neurodegeneration. It is suggested that this process may also contribute to the accumulation of Aβ within the brain [90].

In the studies conducted by Wakatsuki et al., it has been demonstrated that ROS generated by NADPH oxidases in response to damage activate the ubiquitin ligase ZNRF1. This activation triggers intracellular signaling that leads to the degradation of AKT kinase, ultimately resulting in the degeneration of neurites, particularly axons [91,92,93].

Astrocytes actively participate in processes involving the activation of NADPH oxidases. For instance, heightened activity levels of NOX4 induce oxidative stress, mitochondrial fragmentation, and the inhibition of intracellular antioxidant systems in human astrocytes. Moreover, elevated NOX4 levels are likely to promote ferroptosis in astrocytes through oxidative-stress-induced lipid peroxidation, which is associated with impaired mitochondrial metabolism in AD [94].

One potential metabolic pathway for the activation of NADPH oxidases involves the activation of Src family kinases (SFKs) in both neurons and glial cells. The activation of SFKs, such as Fyn, triggers the NFκB-mediated transcription of pro-inflammatory cytokines, iNOS, and gp91phox in microglia and astrocytes. Consequently, the activation of the SFK–iNOS–NOX2 axis results in excessive production of ROS, leading to hyperexcitability and neurotoxicity [95].

Notably, the knockout of the gene encoding p47phox, the organizing subunit of NOX2, has significantly attenuated cognitive impairment and tau pathology in the APP/PS1 mouse model of AD. A p47phox deficiency has also been shown to reduce astrocyte activation. However, it did not affect the Aβ levels and amyloid plaque formation in the brains of aged mice of this strain. Moreover, the neuronal deletion of p47phox was found to mitigate tau hyperphosphorylation at specific sites in primary neuronal cultures following exposure to okadaic acid [96].

It is important to note that there is an ongoing cycle between neuroinflammation and oxidative stress, where one of these pathological processes can trigger the other, creating a positive feedback loop. In summary, astrocytes play a dual role in this process: on the one hand, astrocytes fulfill a pivotal function in antioxidant defense mechanisms, possessing the highest levels of antioxidants among neural cells and offering neighboring neurons substrates essential for antioxidants, including glutathione. On the other hand, reactive astrocytes have demonstrated the capacity to generate diverse free radicals. An augmented expression of −100β and inducible nitric oxide synthase (iNOS) has been associated with oxidative stress, potentially contributing to neuronal demise. 

### 3.4. Interastrocytic Interactions. Calcium Dysregulation in AD

Recent experimental findings suggest that astrocytes form an astrocytic syncytium and can exchange signaling molecules, thereby establishing an astrocyte network [97]. The discovery of calcium waves propagating from one astrocyte to another laid the foundation for the hypothesis that astrocyte networks constitute an extraneuronal pathway for rapid signal transmission across long distances within the CNS [98]. 

A calcium wave denotes a localized elevation in cytoplasmic Ca^2+^ concentration that initiates a sequence of similar events in a wave-like pattern. These calcium waves can be confined to a single cell (intracellular) or transmitted between astrocytes (intercellular) [99]. The primary mechanisms that trigger intracellular Ca^2+^ waves typically involve the activation of G-protein-coupled receptors. This activation leads to the stimulation of phospholipase C and the synthesis of IP3, which binds to IP3 receptors on the endoplasmic reticulum membrane. This, in turn, results in the release of Ca^2+^ into the cytoplasm. Subsequently, intracellular Ca^2+^ waves can propagate to neighboring cells in the form of intercellular calcium waves through two possible mechanisms: Ca^2+^—mobilizing second messengers, predominantly IP3, can be transferred directly from the cytosol of one cell to that of its neighboring cell through gap junctions or by “de novo” generation of such messengers in neighboring cells due to the activation of membrane receptors, which is brought about by the extracellular diffusion of agonists [100].

Gap junctions formed by connexin proteins 30 and 43 (Cx30 and Cx43) are pivotal in the formation of astrocytic conglomerates. These gap junctions facilitate the exchange of ions, metabolites, and neuromodulators, which in turn mediate signaling across the astrocytic network [101]. The expression and functions of connexins, which are responsible for such intercellular communication, have been extensively studied both under normal conditions and in various pathologies, notably in epilepsy and neurooncological diseases [102,103,104,105]. These findings underscore the significance of alterations in the interastrocytic contacts in the pathogenesis of diverse diseases. 

In addition to the established concept of the astrocytic syncytium, current research is actively advancing the hypothesis of astrocytic networks with a more specialized organization [106,107,108]. It is noteworthy that not all neighboring astrocytes exhibit functional connectivity through gap junctions [106,107,109]. This fact could be attributed to the heterogeneous expression of connexins Roux Cx43 and Cx30 in astrocytes or the existence of distinct glial populations emerging from astrocyte distributions within specific spatial domains during development [110]. 

In AD, one of the initial abnormalities observed is the disruption of calcium homeostasis. Specifically, when cultured rat cerebral cortex astrocytes were exposed to Aβ1-42, it resulted in an increase in the amplitude and velocity of the evoked calcium waves and extended the distance over which these waves traveled. These calcium waves were primarily mediated by the increased release of ATP [111]

In vivo multiphoton imaging of high-resolution calcium signaling conducted in the brains of awake APP/PS1 transgenic mice revealed that calcium signaling was disrupted in the thin processes of astrocytes, showing a reduction of about 50%. Conversely, other parts of the astrocyte, including the soma and main processes, displayed hyperactivity, characterized by an increased frequency, amplitude, and duration of calcium signals. Notably, this dysregulation of astrocytic calcium events did not seem to be directly correlated with the proximity to amyloid plaques but rather depended on soluble forms of amyloid [112].

In a recent in vitro study conducted on primary cortical astrocytes, dose-dependent changes in [Ca^2+^]i were observed within a Trimethyltin (TMT) intoxication model. This is a pharmacological model designed to simulate hippocampal degeneration and shares behavioral and molecular characteristics with AD, making it useful for creating an AD-like pathology. Furthermore, the TMT exposure led to mitochondrial depolarization, which occurred independently of extracellular Ca^2+^ and disrupted the antioxidant defense mechanisms in astrocytes, resulting in oxidative and nitrosative stress [113].

Researchers actively investigate the molecular mechanisms responsible for the disruption of calcium signaling. In AD, β-amyloid is known to interact with various receptors, such as the P2Y1R and α7-nicotinic acetylcholine receptors. In the pro-inflammatory environment surrounding amyloid plaques, ATP and ADP are released, activating metabotropic P2Y1 purine receptors (P2Y1Rs) on astrocytes, leading to fluctuations in the calcium levels in astroglia [114]. Additionally, picomolar concentrations of amyloid, which occur in healthy brains in vivo, were found to activate α7-nAChR, resulting in the appearance of calcium waves in astrocytes, which can be considered as a physiological pathway that may play a role in the development of AD [115].

Furthermore, it is plausible that the heightened calcium activity observed in astrocytes could be linked to an increased expression of the metabotropic glutamate receptor mGluR5 in the astrocytes. The activation of mGluR5 has been identified in astroglial cultures exposed to β-amyloid, as well as in astrocytes within animal models of AD and post-mortem human tissues [116,117,118].

One of the non-exocytotic mechanisms contributing to the increased calcium activity in astrocytes and ATP release is connexin hemichannels, specifically connexin 43. In individuals with AD, elevated levels of Cx43 have been observed [119], and exposure to amyloid has been shown to increase Cx43 expression in vitro [120]. In a mouse model of AD, increased Cx43 expression was found to contribute to neuronal damage [121].

An intriguing aspect relates to the role of APOE4, a known risk factor for AD, in calcium signaling disruptions. APOE4 is associated with changes in membrane lipid composition, and lipids are known to regulate Ca^2+^ channels. Raquel Larramona-Arcas et al. demonstrated an increase in ATP-induced Ca^2+^ responses in APOE4 astrocytes compared to APOE3 in male, but not female, mice. This Ca^2^ hyperactivity associated with APOE4 was linked to the dysregulation of Ca^2+^ processing in lysosomes and was mitigated by the expression of APOE3 [122].

Furthermore, beta-amyloid has been found to impact the functioning of calmodulin and numerous calmodulin-binding proteins. Collectively, these factors contribute to the regulation of calcium dyshomeostasis, neuroinflammation, amyloidogenesis, memory formation, neuronal plasticity, and other processes. Detailed insights into the interactions among calmodulin, its binding proteins, and beta-amyloid can be found in the review referenced as [123].

Consequently, virtually all published studies concur that astrocytes in the brains of animal models of AD develop aberrantly increased calcium activity. This stands in contrast to calcium signaling in normal aging, where either no changes or even a reduction in the frequency of calcium events and calcium signaling is observed [124,125]. Notably, this heightened calcium activity in the presence of amyloidosis appears to be unrelated to neuronal activity, as the blockade of neuronal activity with tetrodotoxin fails to normalize spontaneous Ca^2+^ activity [126,127]. 

The optogenetic activation of astrocytes exerts an influence on calcium activity profiles, offering promise in the application of these methods to restore normal astrocytic metabolic activity.

### 3.5. Interaction between Neurons and Astrocytes. Disorders of the Neuron–Astrocyte Interaction in AD. Excitotoxicity

In the mature nervous system, astrocytes establish direct contact with neuronal soma, dendrites, spines, and presynaptic terminals and play an active role in the regulation of neuronal functions [128]. Their functions encompass buffering excess potassium and neurotransmitters in the extracellular space, supplying essential nutrients to neurons, and offering structural support around synapses [129].

Astrocytes are dynamically engaged in synaptic transmission and possess the capability to both respond to neurotransmitters released by neurons and release their own neuroactive substances, known as gliotransmitters (Table 1). Through these gliotransmitters, astrocytes affect the metabotropic and ionotropic receptors on neurons, thereby modulating local synaptic activity [130]. The astrocytic environment surrounding neurons plays a significant role in regulating synaptic connections. For instance, hippocampal neuronal activity has been demonstrated to induce calcium waves within astrocytic networks [131], and astrocyte-generated calcium waves have been shown to modulate neuronal activity [131,132,133,134]. Consequently, neurons and astrocytes engage in bidirectional regulation within neuron–glial networks.

Furthermore, it has been demonstrated that the initiation of astrocyte calcium waves has an impact on synaptic transmission and relies on the regulated exocytosis of glutamate, ATP, and D-serine [135,136,137]. Consequently, these findings introduce novel components, referred to as perisynaptic glia, into the pre- and postsynaptic elements of neuronal transmission, forming a structural entity termed the “tripartite synapse” [128,138,139]. 

Despite the extensive characterization of neuronal and synaptic dysfunctions in AD [140,141], our understanding of the functional impairment of astrocyte physiology, neuronal network function, and astrocyte–neuronal network interactions remains comparatively limited. 

A study conducted by Mitroshina et al. in 2022 aimed to investigate the impact of AD astrocytes on the functioning of healthy neuronal networks in vitro. Their findings revealed that co-culturing healthy neurons with astrocytes treated with Aβ42 resulted in the emergence of aberrant calcium signaling within the neuron–glial network. An increase in calcium signaling was also shown [142].

These findings are consistent with recent research conducted by J. Lines et al. In their study, they employed both calcium imaging and the recording of neuronal electrical activity in APP/PS1 mice. The observed spontaneous hyperactivity of astrocytes was coupled with a diminished responsiveness to sensory stimulation. Furthermore, mice afflicted with AD displayed heightened sensory-induced electrical cortical hyperreactivity, a phenomenon linked to alterations in the interplay between astrocytes and neuronal networks [143].

It can be postulated that the dysfunction of astrocytic networks in AD may disrupt the regulation of cortical electrical activity and contribute to the cognitive decline seen in AD. Reichenbach et al. demonstrated that the pharmacological reduction in astrocyte calcium signaling or astrocyte-specific genetic deletion (Ip3r2−/−) led to the normalization of neuronal network dysfunction and improved spatial memory in the APP/PS1 mouse model [114]. 

Another facet of disrupted calcium homeostasis is the excessive release of excitatory gliotransmitters, a phenomenon that can lead to the development of excitotoxicity [144]. Additionally, impaired glutamate clearance represents another mechanism that can give rise to excitotoxicity and neuronal death. Astrocytes are intricately linked with glutamatergic synapses and are primarily responsible for the extracellular uptake of glutamate [145].

In the early stages of AD development, there is a notable increase in glutamatergic signaling, which is associated with the inadequate clearance of glutamate from the extracellular space. Astrocytes, when interacting with amyloid, lose their capacity to effectively uptake glutamate and cannot maintain synapses [146]. Although the precise mechanisms of these neurological disorders are not fully understood, the dysregulation of GLAST/GLT-1 may play a pivotal role in excitotoxicity and the associated neuropathogenesis. Furthermore, considering that the absorption of glutamate is an energy-intensive process, it is plausible to consider metabolic disturbances within astrocytes that lead to a reduction in this function, ultimately resulting in the excitotoxicity of neurotransmitters. An experiment conducted using a 5xFAD mouse model demonstrated that diminished astrocytic metabolism leads to reduced glutamine synthesis, consequently impairing neuronal GABA synthesis [147].

Summing up, given the significant alterations in astrocyte physiology observed in AD, resulting in disrupted astrocyte–neuronal network interactions, it becomes evident that astrocytes may play a crucial role in the development of cognitive deficits and other neurodegenerative processes associated with AD. It can be confidently stated that targeting the correction of calcium activity within astrocytic networks and addressing other aspects of astrocyte metabolic activity holds great promise as a therapeutic approach.

## 4. Possibilities of Optogenetics for the Treatment of Alzheimer’s Disease

### 4.1. Optogenetic Tools for the Correction of Neurodegenerative Changes in AD

Optogenetic tools have found extensive utility in exploring neural network activity, the roles of various neuronal populations in behaviors, sleep–wake regulation, and are even being investigated for potential applications in the treatment of epilepsy and various neurological disorders. However, there is limited research on the application of optogenetics in AD, particularly in relation to regulating glial cell activity. In this section, we aim to provide an overview of the available evidence in this area.

Among the early investigations in the realm of AD intervention, the study conducted by Iaccarino et al. stands out. In this study, optogenetic stimulation targeted fast-spiking parvalbumin-positive (FS-PV) interneurons at gamma frequencies (40 Hz). Intriguingly, this specific frequency of stimulation, as opposed to other frequencies, demonstrated a capacity to reduce the levels of amyloid-β (Aβ) isoforms, including Aβ 1–40 and Aβ 1–42 [148]. 

Several studies have indicated that the optogenetic activation of hippocampal neurons in models of AD leads to improvements in short-term memory but not in long-term memory. Notably, Cui et al. conducted a study using a pharmacological AD mouse model induced by Aβ injection. They demonstrated that the optogenetic stimulation of ChR2-expressing glutamatergic neurons in the dentate gyrus of the hippocampus resulted in enhanced working and short-term but not long-term memory, led to the activation of mGluR2, a reduction in neuroinflammation, and exerted neuroprotective effects within the central region of ChR2 expression [149]. Similarly, Wang et al. employed a similar AD model and utilized the optical stimulation of glutamatergic neurons with the AAV5-CaMK-CHR2-mCherry construct. Their findings also indicated enhanced short-term memory in response to this stimulation. Furthermore, the activation of GluR2 and mGluR5, an increased expression of the NR subunit of the NMDA receptor, synapsin, and NeuN, as well as a reduced expression of GFAP and IL-6, were observed. These results led the authors to suggest that the activation of glutamatergic receptors, glutamate release, and the modulation of synapsin and NeuN expression levels could potentially underlie the observed memory improvement [150].

In a transgenic mouse model involving APP/PS1 animals, the optogenetic stimulation of pyramidal neurons situated in the CA3 region of the hippocampus effectively restored impaired spatial short-term memory [151]. This intervention was associated with an increase in synaptic density, enhanced synaptic plasticity, and the activation of astrocytes. Of particular significance, when astrocytes were chemogenetically inhibited, the positive effects of pyramidal neuron optostimulation on short-term memory recovery were abolished. This suggests that memory recovery is mediated by the activation of astrocytes [151].

The optogenetic activation of ChR2-expressing astrocytes has been demonstrated to alleviate disturbances in the slow waves of the non-rapid eye movement (NREM) sleep phase observed in APP mice during the early stages of AD progression. It is likely that the correction of slow-wave activity occurred through the normalization of aberrant calcium activity in astrocytes, as astrocytes play an active role in generating slow-wave oscillations in conjunction with neurons. Normalizing these slow fluctuations, typically occurring at frequencies below 1 Hz, is of significant importance since they facilitate long-term plastic changes in neocortical networks and support the consolidation of long-term memory in the neocortex. Furthermore, astrocyte activation led to a reduction in amyloid deposition, prevented elevated calcium levels in neurons, and improved memory performance. However, an important limitation of this study was the continuous optogenetic stimulation over a duration of 14–28 days without consideration of the natural wake/sleep states of the mice [152].

The therapeutic potential of optogenetically activated astrocytes in AD may extend to their ability to regulate the permeability of the blood–brain barrier. In the study by Suo et al. in 2023, the stimulation of astrocytes carrying the GFAP-ChR2-EYFP construct, following the modeling of brain photothrombosis in rats, resulted in a reduction in BBB permeability to immunoglobulins and decreased the formation of gaps in tight junction proteins and the expression of matrix metallopeptidase 2. Additionally, astrocyte photostimulation provided protection against neuronal apoptosis and improved neurobehavioral performance in stroke rats compared to the control group. Notably, the expression of the anti-inflammatory cytokine Il-10 significantly increased in optogenetically activated astrocytes, indicating the activation of neuroprotective type A2 astrocytes. Importantly, inhibiting interleukin-10 in astrocytes counteracted the protective effects of optogenetic stimulation [153].

### 4.2. Application of Optogenetic Approaches to Stimulate Neurogenesis in the Adult Brain in AD

Optogenetic stimulation offers another potentially valuable avenue for addressing neurodegeneration by stimulating neurogenesis. In adulthood, neurogenesis primarily occurs in two main regions: the subgranular zone of the hippocampus (SGZ) and the subventricular zone of the olfactory bulb. This process is crucial for brain plasticity, memory consolidation, and tissue repair following injuries [154]. However, chronic neurodegenerative conditions disrupt neurogenesis. Although the details of neurogenesis are still not fully understood, it is known that early-stage AD hallmarks such as β-amyloid deposition and inflammation hinder the maturation of newly formed neurons and impede hippocampal neurogenesis [155]. Many molecules implicated in AD pathogenesis also play roles in adult neurogenesis regulation. For instance, PSEN1 modulates neural stem cell differentiation in the adult brain by controlling the Notch and Wnt signaling pathways, cleaving the Notch receptor to generate the Notch intracellular domain (NICD) [156]. Meanwhile, sAPPα regulates neural stem cell proliferation [157].

The photostimulation of neural stem cells has been shown to enhance the generation of neuroblasts and functionally competent neurons in vitro by activating the Wnt/β-catenin pathway [158]. The blue light stimulation of ChR2-expressing neural progenitors (NPCs) induced an influx of cations into the cell, resulting in increased progenitor proliferation and differentiation into oligodendrocytes and neurons. Additionally, it led to the transformation of astrocytes from a pro-inflammatory phenotype to a regenerative/anti-inflammatory phenotype [159]. These findings hold great promise for the treatment of neurodegenerative disorders. Moreover, ChR2-expressing neuronal progenitors derived from embryonic stem cells have been successfully transplanted into the mouse cortex. These cells differentiated into GABAergic neurons, exhibited a comprehensive set of neuronal markers, and developed outgrowths and synapses that connected with various brain structures. However, it is worth noting that in vivo photostimulation can yield conflicting results [160]. Various aspects of neurogenesis and optogenetic tools for its stimulation are considered in more detail in the review by Salmina et al., 2021 [161].

Astrocytes play a pivotal role in shaping the microenvironment within the neurogenic niche of the subgranular zone (SGZ) in the dentate gyrus. They release various active molecules and growth factors that initiate and regulate neurogenesis in the adult brain [162]. GFAP-immunopositive radial glial cells located within the neurogenic niches have the potential to give rise to multipotent and dividing progenitor cells. Furthermore, radial glia regulate cell migration, which is crucial for reparative neurogenesis. It is noteworthy that the activation of neurogenesis is always associated with the accumulation of astrocytes in neurogenic niches [163]. Astrocytes express Wnt-3 and promote neuroblast proliferation and the differentiation of neurons into hippocampal granule neurons through the activation of Wnt-mediated NeuroD1 [162,164]. Hence, the expression of light-activated molecules in NSKs under the GFAP promoter can regulate their activity and, accordingly, regulate neurogenesis. For example, the optogenetic activation of ChR2 in astrocytes within an artificial neurogenic niche model has been demonstrated to stimulate neurogenesis [165]. However, it is important to note that there is conflicting evidence suggesting that astrocytes that do not express GFAP and vimentin (GFAP−/−Vim−/−) may have an inhibitory effect on neurogenesis and neuronal differentiation. This inhibition is thought to occur through direct cell-to-cell contact with NSCs and the modulation of the Notch/Jagged1 signaling pathways [166]. Research in this emerging field is still in its early stages, offering ample opportunities for further investigation.

### 4.3. Optogenetics for Modeling AD

Optogenetic approaches have also found application in modeling diseases associated with protein aggregation, including the aggregation of Aβ in AD. An example is the creation of the CRY2-Aβ-mCherry construct, which is based on cryptochrome2 (CRY2) phytochrome derived from Arabidopsis thaliana. This construct exhibits aggregation when exposed to blue light. When Drosophila embryos expressing CRY2-Aβ-mCherry are exposed to blue light, they demonstrate a significant impairment of neuronal function. This optogenetic system has been employed to investigate the effects of signaling pathway activation and drug treatments on Aβ aggregation [167]. Furthermore, this approach has been utilized in model organisms such as Drosophila, C. elegans, and D. rerio to distinguish the detrimental effects of β-amyloid expression and light-induced oligomerization. The damage caused by amyloid-β oligomers closely resembles the pattern of neural tissue destruction characteristic of the late stages of AD [168].

## 5. Problems and Prospects of Using Optogenetics for AD Correction

One significant drawback of optogenetics is its invasive nature, which necessitates the insertion of optical fibers into the brain for light stimulation. Infrared radiation can penetrate tissues more deeply; however, its applicability is constrained by the opsin absorption spectra. Most available opsins respond to blue or green light, which has limited penetration into the brain. Consequently, an implanted light source within the skull with precise placement becomes necessary. A failure to accurately target the desired brain region can compromise the effectiveness of the therapy. 

While this remains a formidable challenge, several research teams have proposed solutions to enable minimally invasive optogenetics. For instance, Chen et al. developed molecularly adapted up-conversion nanoparticles (UCNPs) capable of absorbing near-infrared (NIR) light and emitting visible light. This visible light can be employed to stimulate neurons in deeper brain layers [169]. This represented an initial attempt to devise an optogenetics approach without the need for surgical optical cable implantation into the brain. Nevertheless, the conversion efficiency from near-infrared to blue light is relatively low, potentially necessitating the use of high doses of these particles, which could have neurotoxic effects.

Furthermore, NIR light has demonstrated its impact on cognition by dissociating nitric oxide and enhancing mitochondrial membrane potential, consequently elevating ATP production. Elevated nitric oxide levels can also serve as vasodilators, enhancing nutrient delivery and metabolite clearance [170,171,172,173]. NIR light is employed in photobiomodulation and holds potential as an independent treatment for AD. In a recent development, Gong et al. (2020) introduced a new opsin with ultra-high light sensitivity (SOUL), which, when transcranially optically stimulated, can activate neurons deep within the mouse brain and induce behavioral changes in mice. Encouraging results have also been reported in experiments involving macaques, highlighting SOUL’s high specificity and precise temporal control, rendering it a highly promising tool for the non-invasive optogenetic stimulation of deep brain regions [174]. 

In addition, there is currently a lack of examples showcasing the application of optogenetic methods in the human brain. This absence raises numerous unresolved questions concerning the potential impact of the chosen viral vector on the brain, the ramifications of prolonged light exposure, the development of potential immune responses, toxic effects, and overall safety concerns associated with the technique. An intriguing aspect to consider is that traditional drugs are eventually eliminated from the body, whereas the use of viral vectors for opsin expression implies that these opsins may be expressed for an extended duration, potentially for a lifetime. Thorough investigations are imperative to discern how this prolonged expression might influence the long-term condition of nerve cells. 

When developing optogenetic methods for correcting astrocytic activity, several crucial considerations must be taken into account:

Astrocytes exhibit significant heterogeneity in their inactive and activated states. There are a number of different populations of astrocytes in the brain, differing both morphologically and in their functional and molecular characteristics [53,175,176]. This diversity complicates the design of precisely targeted viral constructs for expressing photosensitive proteins.

In light of the above, it is essential to select stimulation methods and patterns that avoid activating astrocytes along neurotoxic pathways and instead guide their activation toward neuroprotective pathways.

Research is required to evaluate the efficacy of different viral vectors. AAVs of various serotypes, commonly used for neuronal transduction, are not exclusively selective for astrocytes and display varying affinities for different cell subpopulations. AAV5, AAV8, AAV9, or synthetic AAV variants with the desired properties improved through rational capsid engineering are considered optimal for targeting astrocytes. This aspect is considered in detail in the study by Borodinova et al [177].

While a GFAP promoter is frequently used for astrocyte transfection, it is not universal due to the high heterogeneity of the glial population: GFAP is expressed not only in astrocytes, and not all astrocytes express GFAP [178]. Precisely restricting transgene expression exclusively to astrocytes is a challenging experimental task. It may be effective to use, in addition to the main promoters, microRNAs to inhibit this expression in non-target cells [179]. Additionally, the use of specific promoter/serotype combinations appears to be more effective in targeting astrocytes with high efficiency and specificity [177,180].

## 6. Conclusions

Optogenetics has undeniably sparked a revolution in the experimental exploration of neural cell functions and holds substantial promise for addressing neurodegenerative conditions, including Alzheimer’s disease. In this context, optogenetic techniques directed at astrocytes might offer advantages over neuron-centric approaches in ameliorating AD. The identification of optimal protocols for optogenetically stimulating astrocytes holds the potential to mitigate impaired synaptic plasticity, reduce neuroinflammation levels, and restore the integrity of the blood–brain barrier. Nonetheless, in studies involving astrocyte activation, it is imperative to consider the heterogeneity of the astrocytic population and the specific cell types targeted for stimulation.

## Figures and Tables

**Table 1:** Major gliotransmitters.

GABA	Glutamate	Purines	D-Serine
main inhibitory neurotransmitter in the CNS	main excitatory neurotransmitter in the CNS	ATP, adenosine—excitatory neurotransmitters	important in NMDAR modulation
ionotropic GABAA receptors and metabotropic GABAB receptors in neurons	N-methyl-D-aspartate (NMDA) receptors, α-amino-3-hydroxy-5-methyl-4-isoxazolepropionic acid (AMPA) receptors, kainate receptors, and metabotropic receptors in neurons	two families of receptors: P1 (subtypes A1, A2A, A2B and A3), which bind to adenosine, and P2 (ionotropic P2X (seven subtypes P2X1-7) and metabotropic P2Y receptors (8 subtypes)), which are activated by ATP/ADP-nucleotides	a physiological co-agonist of the N-methyl d-aspartate (NMDA) type of glutamate receptor
metabotropic GABA_B_ receptors in astrocytes	glutamatergic transmission within glial cells occurs through metabotropic glutamate receptors (mGluR), which are divided into three groups: group I: mGluR1.5; group II: mGluR2, mGluR3, group III: mGluR4, mGluR6, mGluR 7, mGluR 8	astrocytes express P2X1, P2X2, P2X3, P2X4, P2X5, and P2X7; P2Y1, P2Y2, P2Y4, P2Y6, and P2Y12 receptors; and functional adenosine receptors (A1, A2A, A2B)	
signal transformation from neurons or its amplification through astrocytes depends on the context Under normal conditions, hippocampal astrocytes contain very little GABA	Astroglia regulates extracellular glutamate homeostasis through Na^+^-dependent excitatory amino acid transporters 1 and 2 (excitatory amino acid transporter—EAAT): GLAST-1 and GLT-1, respectively. Astrocytes can release glutamate in a Ca^2+^-dependent and Ca^2+^-independent way. As a gliotransmitter, glutamate can have an inhibitory or excitatory effect on neurons.	ATP released by neurons can affect astrocytes’ purinergic receptors directly in the form of ATP or degradation products in the form of ADP, AMP, and adenosine, leading to an increase in astrocyte Ca^2+^ levels. ATP released from astrocytes is metabolized by extracellular ATPases with the formation of adenosine, which regulates synaptic transmission by affecting the A1 and A2A metabotropic receptors.	a putative gliotransmitter that is associated with learning and memory by affecting synaptic NMDARs
Astrocytes around amyloid plaques become reactive and produce and release GABA aberrantly and in large quantities	In AD, glutamate clearance is impaired. Increased release of glutamate is noted. High spontaneous and abnormal fluctuations in glutamate concentration are observed around Aβ plaques	Enhanced ATP release in hippocampal slices and astrocyte cultures is observed with the application of Aβ peptides	increased in experimental models of AD and in post-mortem samples

## Data Availability

Not applicable.

## Abbreviations

Aβ	amyloid β
AD	Alzheimer’s disease
AMPA receptor, AMPAR	the α-amino-3-hydroxy-5-methyl-4-isoxazole-propionicacid receptor
APOE	Apolipoprotein E
APP	amyloid precursor protein
ATP	adenosine triphosphate
AQP4	aquaporin 4
BBB	blood–brain barrier
ChR2	channelrhodopsin 2
CNS	The central nervous system
CS	complement system
EAAT (GLAST-1 и GLT-1)	the excitatory amino acid transporter
ECB	endocannabinoids
ER	endoplasmic reticulum
EVs	extracellular vesicles
IL	interleukin
IP3	inositol-3-phosphate
HR	halorhodopsin
GABA	gamma-aminobutyric acid
GFAP	glial fibrillar acidic protein
GPCR	G-protein-coupled receptors
LTP	long-term potentiation
MAC	membrane attack complex
MAPT	microtubule-associated protein tau
mGluR	metabotropic glutamate receptors
MMP	matrix metalloproteinases
NFT	нeйpoфибpилляpныe клyбки
NIR	near-infrared light
NSCs	neural stem cells
NMDA receptor, NDMAR	N-methyl-d-aspartate glutamate receptors
PK2	prokineticin-2
PLC	phospholipase C
ROS	reactive oxygen species
SGZ	subgranular zone of the dentate gyrus
TNF	tumor necrosis factor
UDP	uridine triphosphate
